# Macrophage depletion overcomes human hematopoietic cell engraftment failure in zebrafish embryo

**DOI:** 10.1038/s41419-024-06682-x

**Published:** 2024-05-01

**Authors:** Reine El Omar, Naoill Abdellaoui, Safiatou T. Coulibaly, Laura Fontenille, François Lanza, Christian Gachet, Jean-Noel Freund, Matteo Negroni, Karima Kissa, Manuela Tavian

**Affiliations:** 1 University of Strasbourg, INSERM, EFS Grand-Est, BPPS UMR-S1255, Strasbourg, France; 2 University of Montpellier, VBIC, INSERM U1047, Montpellier, France; 3 University of Strasbourg, CNRS, Architecture et Réactivité de l’ARN, UPR 9002 Strasbourg, France; 4 ITI Innovec, Strasbourg, France; 5 AZELEAD SAS, Montpellier, France; 6 University of Strasbourg, INSERM, IRFAC/UMR-S1113, Strasbourg, France; 7 Present Address: Université de Lorraine, CITHEFOR, F-54505 Vandoeuvre Les Nancy, France; 8 Present Address: INSERM, U1256 - NGERE, Université de Lorraine, 54500 Vandoeuvre-lès-Nancy, France

**Keywords:** Haematopoiesis, Myelopoiesis, Phagocytes

## Abstract

Zebrafish is widely adopted as a grafting model for studying human development and diseases. Current zebrafish xenotransplantations are performed using embryo recipients, as the adaptive immune system, responsible for host versus graft rejection, only reaches maturity at juvenile stage. However, transplanted primary human hematopoietic stem/progenitor cells (HSC) rapidly disappear even in zebrafish embryos, suggesting that another barrier to transplantation exists before the onset of adaptive immunity. Here, using a labelled macrophage zebrafish line, we demonstrated that engraftment of human HSC induces a massive recruitment of macrophages which rapidly phagocyte transplanted cells. Macrophages depletion, by chemical or pharmacological treatments, significantly improved the uptake and survival of transplanted cells, demonstrating the crucial implication of these innate immune cells for the successful engraftment of human cells in zebrafish. Beyond identifying the reasons for human hematopoietic cell engraftment failure, this work images the fate of human cells in real time over several days in macrophage-depleted zebrafish embryos.

## Introduction

Hematopoietic stem cells (HSC) are self-renewing multipotent cells capable of producing all blood cell types during the entire life of an individual. Clinically, these cells are the relevant component of bone marrow transplants, used to treat patients with blood disorders and malignant diseases [1].

Data on human HSC characterization and potentialities have been collected mainly through in vitro assays and by tracking in vivo the fate of transplanted bone marrow cells in various wild-type and humanized animal models [2–6]. Successful transplantation requires the ability of HSC to migrate toward suitable niches as well as to integrate signals that promote their self-renewal and differentiation [7].

Historically, mouse has been viewed as the “gold standard” for xenotransplantation experiments. Nonetheless, this model has major limitations. For instance, the direct imaging of engrafted cells is limited even by using highly sensitive bioluminescent techniques; mouse xenograft experiments need to use immunocompromised animals to prevent early rejection of human transplanted cells by the mouse immune system; and the monitoring of these immunodepleted mice after transplantation is tedious and requires a relatively high number of grafted cells and elevated costs.

The use of zebrafish (*Danio rerio*) as an alternative research organism has grown rapidly over the last decades [8]. The zebrafish genome contains orthologues to ~70% of human genes, and 80% of those are involved in diseases [9]. Many characteristics make the zebrafish a powerful model, such as short generation time, high fecundity, external embryogenesis and fast development stage. In addition, zebrafish embryos are optically transparent allowing direct noninvasive live imaging with great sensitivity reaching the single-cell level, generally not achievable in mouse [10]. Moreover, given that adaptive immunity in zebrafish does not reach maturity until 4 weeks post fertilization [11], cell graft-versus-host rejection can be circumvented using animals at early developmental stages [12, 13]. Thus, zebrafish embryo xenograft models have been taken advantage of to study several types of solid cancers [13, 14].

Despite the bulk of studies reporting the feasibility of xenografting solid tumor cells into zebrafish, only few data exists for human malignant hematopoietic cells and even less for healthy hematopoietic progenitors [15–20] even though human and zebrafish hematopoietic system shares many features. Indeed, in both cases cells with HSC potential first emerge in ventral wall of the aorta through an endothelial-to-hematopoietic transition, then they migrate to a transient site of hematopoiesis, the caudal hematopoietic tissue (CHT) in zebrafish and the liver in mammals, before colonizing the thymus and pronephros in zebrafish (kidney marrow) and the bone marrow in mammals [21, 22]. Moreover, we and colleagues have demonstrated that the signaling pathways regulating hematopoiesis are highly conserved between zebrafish and mammals [23, 24]. The transparence of zebrafish embryos has further allowed to visualize in situ the emergence, migration and behavior of emerging blood cells [25].

Recently, it has been shown that human HSC do not survive > 24 h when transplanted into wild-type zebrafish embryos and only 48 h in transgenic embryos humanized to express human cytokines such as CXCL12, SCF, and GM-CSF [26, 27]. The reason for the short-term survival of transplanted cells in zebrafish embryos remains elusive but the adaptive immune system could be ruled out because it does not become fully functional before 4 weeks after fertilization.

In the present study, we have addressed this issue by developing a rapid method of labeling of human cells that were then transplanted and followed in zebrafish embryos. We uncovered the major role played by elements of the innate immune system, the macrophages, in the short-term survival of transplanted cells and we developed a new normalized protocol allowing the long-term survival of human hematopoietic transplanted cells.

## Results

### Lentivirus transduction and spinoculation: transduction efficiency in human hematopoietic CD34+ cells and leukemia Jurkat cells

The key factor for xenotransplantation assays is distinguishing the transplanted cells from host cells. Cell membrane stains have been extensively developed due to their ease of use, their rapid visualization, and the large array of colors they provide. However, depending on the cell type and the concentration required for stable labelling, they can present high toxicity. Moreover, the concentration of these stains inside the cell decreases as cells divide, and the stains can be released into the tissue and be transferred to other cells compromising the reliability of in vivo live imaging [28]. Thus, we choose to label cells by endogenously expressed green fluorescent protein (GFP). Lentiviral vectors (LV) are frequently used for delivering, upon transduction of the desired cells, a transgene of interest to human cells, both proliferating and quiescent. By integrating the transgene in the genome of the transduced cell, LV lead to stable labelling of the cell and its descendance [29].

**Fig. 1:**
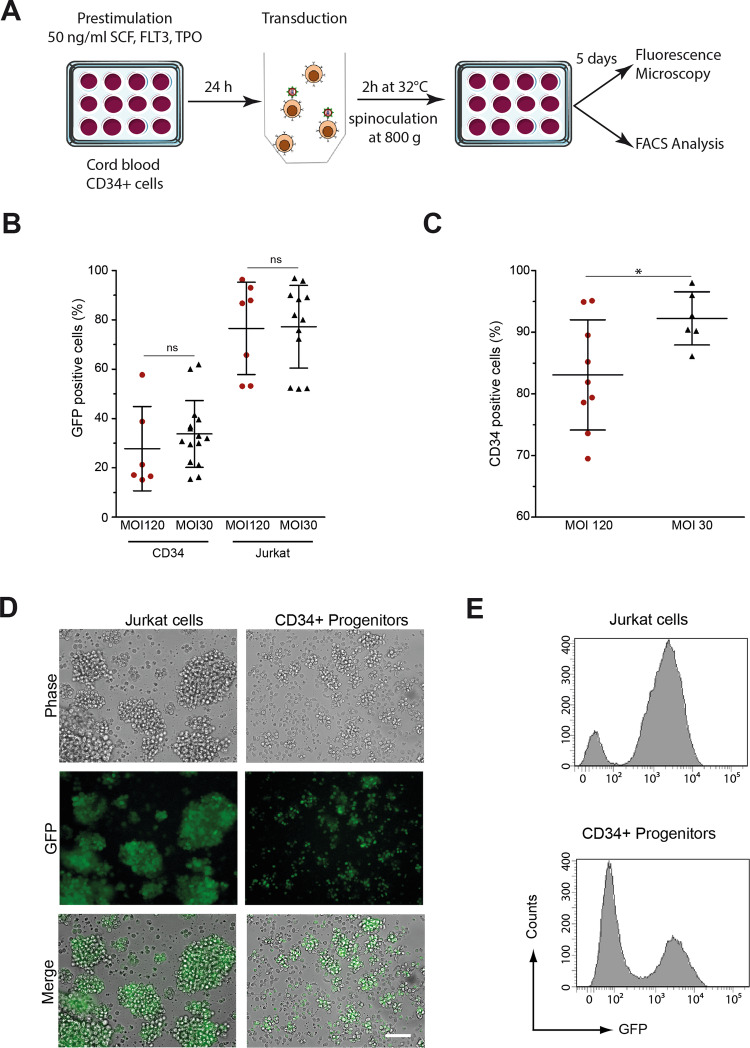
Transgene transfer and GFP expression after transduction of human CD34+ and Jurkat cells. **A** Human CD34^+^ blood cells from umbilical cord blood and Jurkat cells were transduced by spin inoculation with lentiviral vectors encoding a nuclear variant of the eGFP at multiplicity of infection (MOI) of 30 and 120 and cultured in vitro. After 5 days, cells were analyzed for GFP expression by both flow cytometry and fluorescence microscopy. **B** Estimates of the efficiency of transduction based on the percentage of GFP^+^ cells. **C** Percentage of cells expressing CD34 after 5 days of culture. **D**, **E** A representative experiment showing GFP expression after transduction of human CD34^+^ or Jurkat cells at the MOI of 30. In (**E**), results are represented as histograms of GFP fluorescence intensity (*x*-axis, log scale) versus cell number (*y*-axis, linear).

We thus developed a rapid and reliable method for fluorescent staining of hematopoietic cells based on lentiviral transduction. For this purpose, human FACS-sorted CD34^+^ cells were prestimulated with cytokines for 24 h and then transduced using the spin inoculation method with an HIV1-based lentiviral vector expressing a nuclear version of the enhanced green fluorescent protein (nGFP) (Fig. 1A). The use of the nuclear version of eGFP results in a brighter and more focused signal than its cytoplasmic version, facilitating the following of the labelled cells in the subsequent steps of the experiments. The Jurkat cell line was used as positive control for transduction, and cord blood was the source of CD34^+^ normal hematopoietic stem/progenitor cells. Two multiplicities of infection (MOI) were tested: 30 and 120. Both MOIs yielded comparable percentages of transduced cells with values of 33.8 ± 3.5% in cord blood CD34^+^ cells and of 77.2 ± 4.8% in Jurkat cells (Fig. 1B).

Importantly, though, for the MOI of 30, the expression rates of CD34 remained significantly higher than for the MOI of 120 over time, as determined at day 5 in culture after transduction of CD34^+^ cells (92.2 ± 1.8%, at MOI 30 vs 83.1 ± 3.0% a MOI 120, *p* < 0.05) (Fig. 1C), indicating that the low MOI preserved a stable stem/progenitor phenotype of human CD34^+^ cord blood cells. Transduced CD34^+^ and Jurkat cells retained a normal morphology (Fig. 1D) and expressed a high level of nGFP confirmed by flow cytometry (Fig. 1E), which allowed easy visualization once transplanted into zebrafish embryos (see below). Therefore, based on previously described techniques of spin-inoculation [30], and on a MOI of 30, which is 3 – 100 times lower than those used in previous studies [31, 32], we document that only 2 h of incubation with our vectors are sufficient for yielding an efficiency of transduction comparable to the one obtained with longer exposure time to the vectors described in other studies [32, 33]. Shortening times of transduction by spin-inoculation provides the potential to reduce ex vivo manipulation of the target cells with the aim of preserving their functions and undifferentiated state before transplantation.

### Fates of transplanted hematopoietic cells into zebrafish embryos

Transduced Jurkat (JK-GFP) and CD34^+^ hematopoietic (CD34-GFP) cells (50–200) were transplanted in the caudal vein of wildtype (WT) zebrafish embryos, at 36 h.p.f. stage. The cell distribution in embryos was checked immediately after injection and then followed over time.

### Survival, proliferation and homing of transplanted human cells

**Fig. 2:**
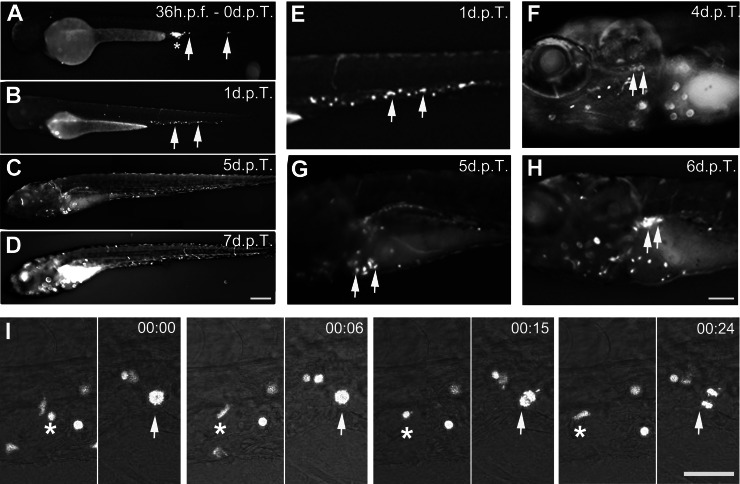
Transplantation of human JK-GFP cells in zebrafish embryos, colonization of hematopoietic organs and proliferation. **A** Transplantation of JK-GFP cells in the caudal vein of a wildtype zebrafish embryo at 36 h.p.f. **B**–**D** Colonization of zebrafish hematopoietic tissues followed from 1 to 7 d.p.T. Colonization of the CHT (**B**, **E**, arrows), thymus (**F**, arrows), fetal liver (**G**, arrows), kidney (**H**, arrows). See Video 1. **I** Live imaging of JK-GFP cells circulation (asterisk) and proliferation (arrow) in the CHT (Time code 00:15 and 00:24). See Video 2. CHT: Caudal Hematopoietic Tissue. Scale bars: (**A**–**D**): 250 µm; (**E**–**H**):100 µm, (**I**): 25 µm. Time code in minutes.

In all transplanted embryos, right after injection, JK-GFP cells were mostly localized at the injection site (*n* = 92) (Fig. 2A asterisk), while few cells were also found in the caudal hematopoietic tissue (CHT) (Fig. 2A and Fig. S2A, B arrows). At 1 day post-transplantation (d.p.T.), JK-GFP cells were mainly distributed in the CHT (Fig. 2B and E, and Fig. S2C–D arrows) (70/92). In rare transplanted embryos (2/92), from 2 d.p.T. to 7 d.p.T., fluorescent cells actively circulated in the vascular system of zebrafish embryos (Fig. 2C, D and Video 1), extravasating the blood vessels and colonizing the different hematopoietic organs: thymus (Fig. 2F, arrows), fetal liver (Fig. 2G, arrows) and kidney (Fig. 2H, arrows). These results confirmed, as previously described, that transplanted human Jurkat cells can survive in the zebrafish embryo [19]. Moreover, we observed an slight increase with time of the number of GFP-cells accumulating within the CHT of embryos (Fig. 2A–D). Indeed, quantification of GFP fluorescence as described in Supplementary Material showed a reduction of JK-GFP cells at 1 d.p.T. (79%), followed by a stabilization phase in cell numbers at 4 d.p.T. and then a steady growth at 5 (131%), 6 (145%) and 7 d.p.T. (166%) compared with the number of cells quantified at 0 d.p.T.

In line with this, following the behavior of injected cells at single cell level by live imaging highlighted that JK-GFP cells were able to migrate (Fig. 2I, asterisk) and undergo cell division (Fig. 2I, arrow), indicating that the progressive increase in the number of fluorescent cells was due to cell proliferation (Fig. 2I and Video 2). Together, these observations revealed that the environment of the zebrafish embryo provides efficient signals allowing survival, proliferation and homing of human hematopoietic cells into the hematopoietic organs.

### Engraftment failure and transplanted cell disappearance

Although the above experiments with leukemia cells (JK-GFP cells) demonstrate the feasibility of human hematopoietic cells engraftment, proliferation, and colonization of hematopoietic organs in WT zebrafish embryos for up to 7 d.p.T., the success rate of the process was very low (2/92) and it was never observed with primary human CD34-GFP cells (*n* = 124) that survive only 2 days after injection within the zebrafish embryo (Fig. S2M, arrow and Video 3), as previous described [19, 27]. Indeed, in the large majority of injected embryos, the number of human transplanted cells, either JK-GFP or CD34-GFP, decreased dramatically few hours after the injection, to disappear in the next days (Fig. S2E–H and N), even if rare cells (1–5 per embryo) could survive up to 3 days and 7 days after transplantation for CD34-GFP and JK-GFP cells, respectively, if they had reached the CHT (data not shown).

**Fig. 3:**
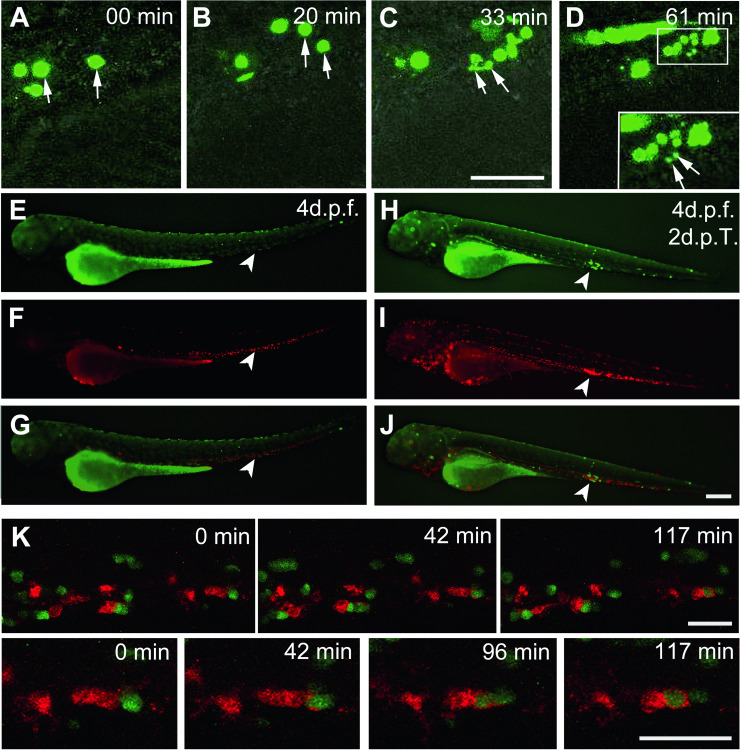
Human JK-GFP cells phagocytosis by zebrafish embryonic macrophages. **A** Live imaging of JK-GFP cells injected in a zebrafish embryo at 30 h.p.f. (arrows indicate two cells). Starting from 33 min post transplantation, some green cells appear fragmented (**C**, **D** arrows). **D** Inset shows a high magnification of fragmented cells at 61 min (arrows). Each frame is a maximum projection of 3 planes apart 1 μm. See also **Video 4**. **E**–**J** Macrophages recruitment in a 4-d.p.f. *Tg (mpeg1:mCherry)* zebrafish embryo after injection with PBS (**E**–**G**) or with JK-GFP cells (**H**–**J**) (arrow). Merged picture (**J**) shows the accumulation of macrophages around JK-GFP cells. **K** Live imaging of mCherry-macrophages in a 4-d.p.f. embryo, 2 days after transplantation of JK-GFP cells. High magnification reveals the phagocytosis of green human cells engulfed by red host macrophages. Each frame is a maximum projection of 5 planes apart 0.8 μm.See also **Video 5**. Scale bars: (**A**–**D**): 25 µm, (**E**–**J**): 250 µm, (**K**): 20 µm. Time code in minutes.

To understand the origin of this early disappearing, we imaged in real time the fate of JK-GFP cells after injection. Remarkably, in the minutes following transplantation, human cells were lysed (Fig. 3A–D, arrows). Closer examination using live confocal microscopy showed that cells were fragmented before disappearing (Fig. 3D, 61 min, inset; Video 4). Since primitive macrophages are already present in the zebrafish embryo at the developmental stage used for human cell transplantation [34], we speculated that this cell type of the innate immune system could be responsible for the clearance of human transplanted cells.

### Phagocytosis by zebrafish embryonic macrophages at the origin of transplantation failure

**Fig. 4:**
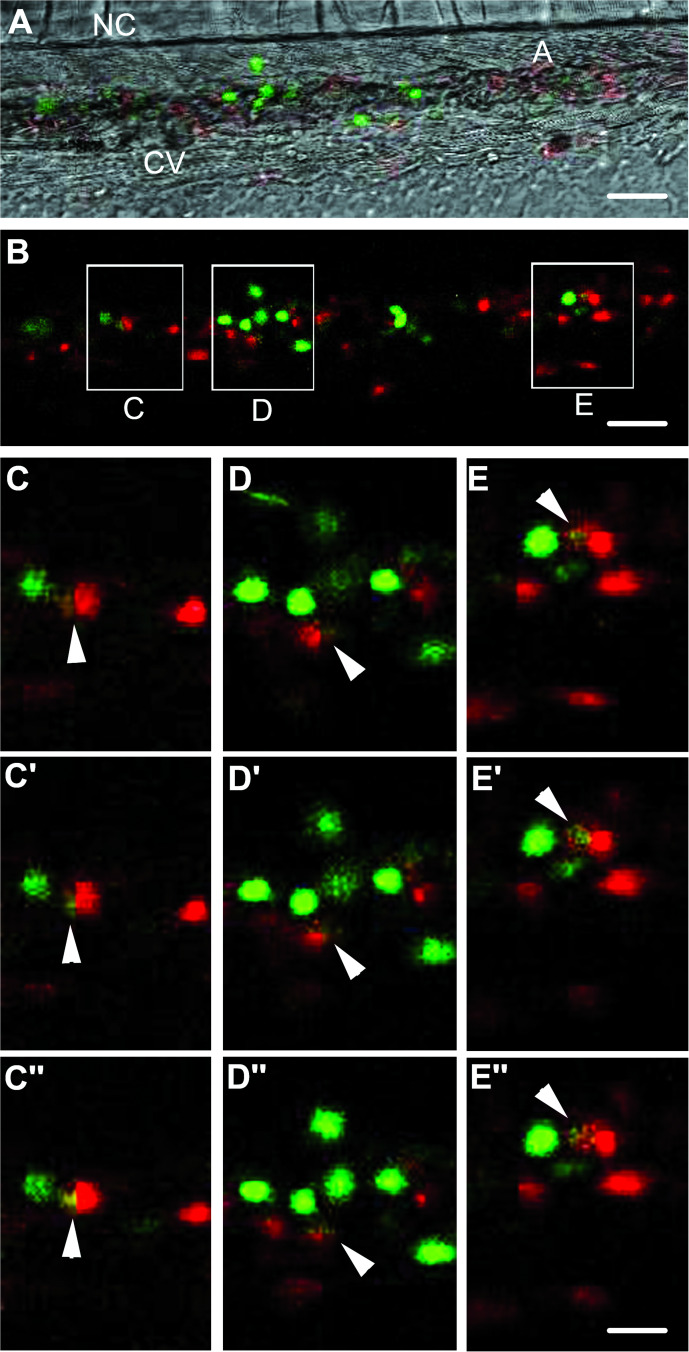
CD34-GFP cells phagocytosis by zebrafish embryonic macrophages. **A**, **B** Z-stack confocal imaging of mCherry-expressing macrophage behavior in a zebrafish embryo 2 h post transplantation (h.p.T.) of CD34-GFP, reveals the accumulation of macrophages around green cells and their phagocytosis (inset **C**, **D** and **E**). **A** Bright field imaging of caudal hematopoietic tissue in the tail region at 30 h.p.f., merged with confocal fluorescent imaging of CD34-GFP cells and mCherry-expressing macrophages (**B**). **C**–**E”** 3 successive z-stacks confocal planes apart 0.8 μm of phagocytosis of CD34-GFP cells by mCherry-expressing macrophage (arrows). See also **Video 6**. A aorta, CV caudal vein, NC notochord. Scale bars: **A**, **B**: 25 µm; **C**–**E**”:10 µm.

To look whether primitive embryonic macrophages were involved in human cell lysis and transplantation failure, JK-GFP cells were injected in Tg(*mpeg1:mCherry*) zebrafish embryos. In this line, the Mpeg1 promoter drives the specific expression of membrane targeted red-fluorescent protein in macrophages, allowing their tracing in vivo [35]. With these *Tg(mpeg1:mCherry)* embryos, we analyzed the distribution of macrophages at 4 days post fertilization (d.p.f.) after either injection of PBS (Fig. 3E–G) or JK-GFP cells (Fig. 3H–J). In vivo imaging showed an important recruitment of macrophages at the site of transplantation of JK-GFP cells (Fig. 3I, arrowhead) unlike the site of injection with PBS (Fig. 3F, arrowhead). Moreover, the simultaneous visualization of red-macrophages and JK-GFP cells revealed co-localization of both cell populations (Fig. 3J, arrowhead). Time-lapse confocal imaging, between 0 and 15 h post transplantation (h.p.T.) showed that primitive macrophages of zebrafish embryos were able to efficiently phagocyte the JK-GFP cells (Fig. 3K, and Video 5). Same behavior was observed after transplanting human CD34-GFP cells (Fig. 4, and Video 6), which completely disappeared just few hours after injection (Fig. S2N).

### Improved cell survival with macrophages escape

**Fig. 5:**
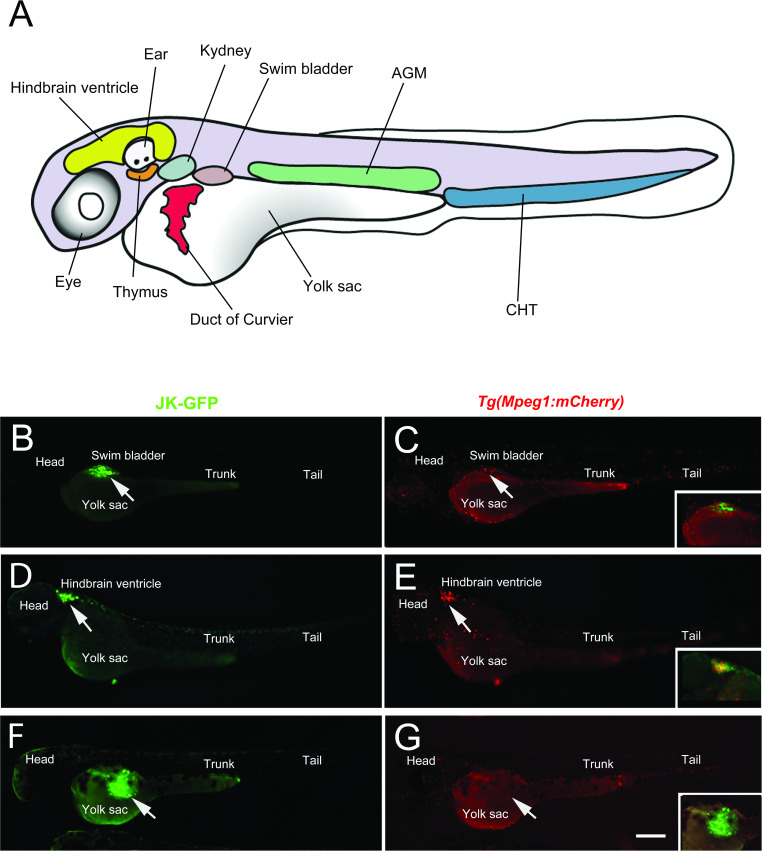
Comparison of injection sites in the zebrafish embryo. **A** Different injection sites of zebrafish larvae used in this study. Injection of JK-GFP cells in swim bladder (**B**, **C**), hindbrain ventricle (**D**, **E**) and yolk sac (**F**, **G**), of 30 h.p.f. *Tg(mpeg1:mCherry)* zebrafish embryos. Merged pictures in the insets show macrophages accumulation at 6 h.p.T. in the swim bladder (**C**) and the hindbrain ventricle (**E**) but not in the yolk sac (**G**). Arrows indicate the site of injection. AGM aorta-gonad-mesonephros, CHT caudal hematopoietic tissue. Scale bar: 250 µm.

Next, we investigated if there is a relationship between the efficacy of human hematopoietic cell engraftment and the capacity of macrophage recruitment at the site of cell injection, while searching for a site where macrophages would be absent or poorly recruited. For this purpose, we first injected 100–200 JK-GFP cells in the nascent swim bladder of 30 h.p.f. Tg (*mpeg1:mCherry*) zebrafish embryos (Fig. 5A, B) (*n* = 10). Six hours after injection, an extensive recruitment of macrophages was observed at the injection site (Fig. 5C, arrow) compared to the injection with PBS (Fig. S3, arrow) followed by the disappearance of human cells in the next days (data not shown). Same results were obtained when fluorescent cells were injected in the hindbrain ventricle of 30 h.p.f. *Tg(mpeg1:mCherry)* embryos (*n* = 10) (Fig. 5D, E and Fig. S3, arrow), indicating that neither of these sites was compatible with efficient survival and growth of human hematopoietic cells.

The yolk sac (YS) is the most common location used for injection of human cells from various tissue origin in zebrafish embryos. The interest of this site lies in its large size allowing easy transplantation of numbers of cells [13], and its acellular, partly avascular and nutrient-rich environment which supports cell proliferation [12]. Taking this into consideration, we analyzed the accessibility of the YS to macrophages after transplanting transduced cells in 30 h.p.f. *Tg(mpeg1:mCherry)* zebrafish embryos. Unlike the swim bladder and the hindbrain ventricle, no macrophage was detected into the YS 6 h after cells injection (Fig. 5F, G), and a mass of both JK-GFP and CD34-GFP cells was recovered into the YS at 1 d.p.T (Fig. S4A–D). The results were confirmed by a quantification of green fluorescent pixels. JK-GFP cells grew by 135.4% (*n* = 6) and CD34-GFP cells by 105.1% (*n* = 6), 1 d.p.T. in the YS.

Taken together, these observations suggest that the absence of macrophages correlates with survival and maintenance of human hematopoietic cells in the zebrafish embryo. However, since injected cells remain sequestered in the YS and failed reaching the blood circulation, the use of this site of injection cannot fulfill the requirements for studies of the hematopoietic system and its pathologies. Therefore, we searched for an alternative way to avoid macrophages.

### Macrophage depletion allows human hematopoietic cell xenograft success

To precisely demonstrate the involvement of macrophages in xenograft failure, we genetically and pharmacologically depleted primitive macrophages before cell engraftment.

**Fig. 6:**
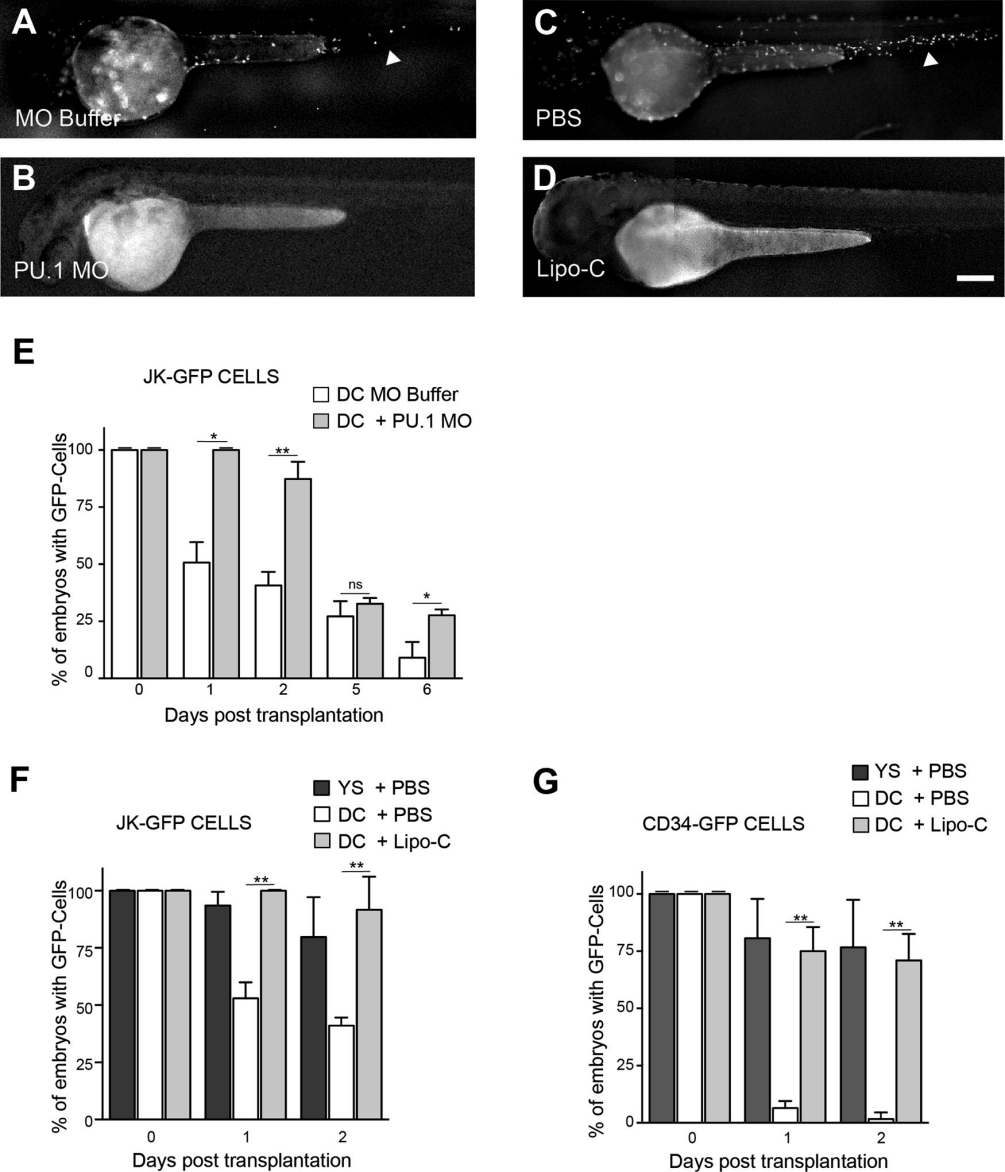
Genetic and chemical depletion of macrophages in zebrafish embryos. Primitive macrophages were genetically and chemically depleted before cell engraftment in Tg (*mpeg1:mCherry*) zebrafish embryos. **A**–**D** Complete and efficient depletion of macrophages is observed in PU.1 morphant (**B**) and in Lipo-C-treated (**D**) embryos at 35 h.p.f and 48 h.p.f, respectively, compared to controls (**A** and **C**) which contain fluorescent macrophages (head arrows). **E** JK-GFP cells injected in PU.1 treated and non-treated zebrafish embryos are followed for 6 days.  Injection into the Duct of Cuvier (DC) of MO-buffer-treated embryos.  Injection into the Duct of Cuvier (DC) of PU.1 Morphant embryos. **F**, **G** Transplantations of JK-GFP (**F**) and CD34-GFP (**G**) cells in the Duct of Cuvier (DC) of Lipo-C-treated embryos show a percentage of engraftment comparable to that obtained with injections into the yolk sac. Scale bar: 250 µm.  Injection in the yolk sac of PBS-treated embryos.  Injection into the Duct of Cuvier (DC) of PBS-treated embryos.  Injection into the Duct of Cuvier (DC) of Lipo-C-treated embryos. Data were presented as mean ± SD and are representative of 3 independent experiments with *n* = 15–20 (E), *n* = 10–30 (**F**) and *n* = 10–30 (**G**) embryos/group. (ns not significant or *p* ≥ 0.05; *p < 0.05; **p < 0.01; ***p < 0.001; ****p < 0.0001).

First, we used a genetic approach by injecting antisense morpholino against the myeloid transcription factor gene *PU.1*, required for differentiation of both primitive macrophages and neutrophils [36]. PU.1 morpholino (PU.1 MO) led to complete depletion of macrophages the day after treating embryos (Fig. 6B), compared to controls (Fig. 6A). Then, when JK-GFP were intravenously injected in the duct of Cuvier (posterior cardinal vein) of PU.1 morphant *Tg(mpeg1:mCherry)* embryos at 24 h.p.f, 100% of embryos (24/24) contained transplanted cells at 1 d.p.T, unlike controls, where JK-GFP cells were detected in only 51% of PU.1-expressing embryos (16/30) (Fig. 6E). At 2 d.p.T, the percentage of PU.1 MO-treated embryos exhibiting JK-GFP decreased to 87% (19/22) vs 41% for the control group (12/29), while the percentage of embryos still containing JK-GFP cells was similar (≈30% on average) in both groups at 5 d.p.T. Thus, macrophages depletion by PU.1 MO significantly improved the transplanted cells uptake and survival for the first 2 d.p.T., but the effect was transient because macrophages progressively reappeared with time in PU.1 morphant embryos (Fig. S5H) indicating the rescue of myeloid development (Fig. 6E).

To strengthen these results and run out the possibility that neutrophils depletion also occuring using PU.1 morpholino could be responsible of the survival of transpanted cells, we used a chemical approach with Liposome encapsulated Clodronate (Lipo-C), well described to specifically deplete already existing phagocytosing macrophages [37].

Corroborating our previous observation [38], injection of Lipo-C into the caudal vein of 24 h.p.f. Tg(*mpeg1:mCherry*) zebrafish embryos induced efficient macrophages depletion (Fig. 6D), compared to embryos injected with PBS (Fig. 6C). The specificity of this approach in macrophage but not neutrophil depletion has been further confirmed using the double transgenic *Tg(mpx:eGFP)/Tg(mpeg1:mcherry)* zebrafish (Fig. S6E–H). When *Tg(mpeg1:mCherry)* embryos were intravenously transplanted in the duct of Cuvier at 30 h.p.f. with JK-GFP cells, Lipo-C treatment allowed maintaining of 92% of injected cells (42/46) at 2 d.p.T., unlike the PBS-treated embryos where this percentage fall to 41% (16/39) (on average in 3 independent experiments, 10 to 30 embryos/group) (Fig. 6F). Similar results were obtained with human CD34-GFP cells intravenously transplanted at 30 h.p.f. in the duct de Cuvier of respectively Lipo-C and PBS-treated *Tg(mpeg1:mCherry)* embryos (Fig. 6G). At 2 d.p.T., the percentage of embryos containing CD34-GFP cells drastically decreased in the PBS-trated group (2%, 1/50) but remained elevated in the Lipo-C treated group (71%, 30/37), on average in 3 independent experiments, 10–30 embryos/group (Fig. 6G). Interestingly, these results correlated with what has been shown when hematopoietic cells were injected into the YS, a condition where transplanted cells are isolated from the zebrafish macrophages (Figs. 6F, G and S4).

## Discussion

Several studies have documented the xenotransplantation of human leukemic cell lines, primary leukemic cells and other cancer cell types in zebrafish embryos, indicating that immaturity of the adaptive immune system at this stage is crucial for efficient xeno-engraftments [reviewed in [13, 39]]. However, engraftment of human blood stem cells remains a challenge [27, 40, 41]. Indeed, although human blood CD34^+^ cells have been transplanted in immunocompetent zebrafish, they completely disappear 24 h.p.T. [26, 27]. Recently, progress toward longer engraftment used genetically modified animals lacking adaptive immune cells [42], which however still have limitations, as they require complex husbandry approaches [43]. Furthermore, given that zebrafish embryos still lack mature adaptive immunity, the mechanisms for successful engraftment of human cells in these immune compromised animals remain unexplained [42].

In the present study, we demonstrate that macrophages, a component of innate immunity, is an important determinant of xenograft failure as the depletion of these cells significantly improves early survival of immortalized as well as primary human hematopoietic cells transplanted in zebrafish embryos. To reach this conclusion, we first developed a rapid and efficient fluorescent labelling procedure of human CD34^+^ cells based on lentivirus infection allowing long term stem cell staining without major alterations of the properties of transduced cells.

Our findings show that depending on the cell type, either normal (primary CD34^+^ cells) or cancerous (Jurkat cells), and on the injection site, cells disappear rapidly after injection into zebrafish embryos or survive and proliferate. Indeed, human CD34^+^ cells are detected only at 1 d.p.T. and disappear rapidly, which is consistent with what was reported by other authors for primary cells [19, 27], whereas Jurkat cancer cells can be detected in the circulation up to 7 d.p.T. These cells circulate actively in the vascular system of the zebrafish embryo, proliferate and colonize hematopoietic niches indicating that the zebrafish hematopoietic environment provides efficient signals for proliferation and homing of human leukemia cells. Intriguingly, we observe rare CD34^+^ cells also surviving for up to 2 days when they are niched in the perivascular pockets into the CHT. In agreement with a previous report showing that human CD34^+^ cells transplanted into zebrafish home to the CHT and respond to zebrafish stromal-cell derived factors [41], we speculate that cells are protected in these perivascular pockets.

In zebrafish, macrophages are the first leukocytes appearing in the developing embryo. They are detected as early as 20 h.p.f. at the surface of the YS, then migrate into the mesenchyme to colonize all the embryonic organs [34]. These cells display an important phagocytic activity against pathogens, assuring an efficient innate immune response, and play important roles in development and tissue homeostasis [38, 44–46].

In this work, convergent observations support the primary role played by macrophages in transplantation failure of human hematopoietic cells in zebrafish embryos. First, we show that human engrafted cells induce extensive recruitment of primitive macrophages at the site of injection that rapidly phagocyte the transplanted cells. Second, engraftment efficacy inversely correlates with the recruitment of macrophages in contact with transplanted cells. Indeed, engraftment of human hematopoietic cells is efficient into the YS preserved from macrophage entry, but not in other sites such as the swim bladder, the hindbrain ventricle or the duct of Cuvier where macrophages can reach the xenotransplanted cells. Third, macrophage depletion using genetical or pharmacological approaches improves early engraftment of the human hematopoietic cells. Moreover, as long as macrophages are absent, transplanted human cells can survive in the zebrafish embryos showing that the zebrafish environment is suitable for the survival of human hematopoietic cells.

To our knowledge, the present study documents for the first-time, using a combination of real-time imaging and functional assays, the importance of primitive macrophages in the failure of xenotransplantation of human hematopoietic cells in the widely-used model of zebrafish embryos, at a stage when adaptive immunity is not yet active. Indeed, although the adaptive immune system is absent at early developmental stages, successful xenotransplantation requires to control the primitive wave of hematopoiesis producing myelo-eryhtroid cells, specifically macrophages that appear as early as 20 h.p.f. [34].

Thus, this study combined with previous works [26] suggests that efficient engraftement of human primary hematopoietic cells would need to combine primitive macrophage depletion to prevent cell disappearance with the providing of human cytokines to sustain cell survival and proliferation.

## Material and methods

### Umbilical cord blood CD34+ cells sorting

Umbilical cord blood (UCB) units were collected from full-term deliveries after receiving a written consent, according to the guidelines of the French National Ethics Committee. The study has been approved by the IRB Institutional of the French Institute of Medical Research and Health (Number 21-854). UCB was processed within 24 h after reception. Mononuclear cells (MNC) were isolated by Ficoll-Histopaque (1077 g/ml) (10771-500 ML, Sigma-Aldrich) density centrifugation. Low-density cells were washed twice in Dulbecco’s Phosphate-Buffered Saline (PBS, 14190-094, Gibco) supplemented with 2% fetal calf serum (FCS) (P30-3302, Pan Biotech) and then processed for CD34^+^ cells sorting. Briefly, MNC were resuspended in PBS containing 2% FCS and incubated with FITC mouse anti-human CD45 (Clone 5B1, 130-080-202, Miltenyi) and APC mouse anti-human CD34 (clone AC136, 130-090-954, Miltenyi) for 30 min at 4 °C. Cells were then washed with 2 ml of PBS 2% FCS and resuspended in a solution of 7-Aminoactinomycin (7-AAD, 2 µg/mL) (A9400, Sigma-Aldrich). Cell sorting was performed on a BD Aria II flow cytometer. Sorted CD34^+^ cells were reanalyzed to establish purity (>95%). Isolated CD34^+^ cells were cultured in serum-free expansion medium consisting of StemSpan (09650, StemCell Technologies) containing recombinant human Stem Cell Factor (SCF, 50 ng/ml, 255-SC-050, R&D Systems), recombinant human Thrombopoietin (TPO, 50 ng/ml, 288-TP-025, R&D Systems) and recombinant human Flt-3 Ligand (Flt-3l, 50 ng/ml, 308-FK-025, R&D Systems). Cells were incubated overnight at 37 °C with 5% CO_2_ and then transduced by lentiviral vector.

### Cell lines

Cell lines were obtained from the American Type Culture Collection (ATCC). HEK 293 T were cultured in Dulbecco’s Modified Eagle’s Medium (11885-084, Gibco) supplemented with 10% FCS and 1% penicillin/streptomycin. Jurkat cells were cultured in Roswell Park Memorial Institute medium (11875-093, Gibco) supplemented with 10% FCS and 1% penicillin/streptomycin. Cells were maintained at 37 °C with 5% CO_2_.

### Construction of plasmid pSDY-nGFP

pSDY-nGFP was obtained by replacing, in the pSDY-dCK-Puro plasmid [47], the sequence encoding human deoxycytidine kinase by a cassette with the sequence for eGFP (nt 75 – 453 of the homo sapiens cDNA FLJ9211; Genebank AK311849.1) preceded by the nuclear localization signal of histone H2B (nt 46-423, accession number NM_021058).

### Viral vector production and titer

For lentiviral vector production, 5 × 10^6^ HEK 293 T cells were plated in 10 cm diameter plates and transfected at 80% of confluence with a mix containing 2 ug of pCMVΔR8.91 [48], 1ug pHCMV-G (Yee JK et al, PNAS, 1994; 91:9564–8), 2 ug of pSDY-nGFP, Opti-MEM (Gibco, thermo Fischer scientific, Waltham, MA, USA) and polyethylenimine (Polysciences, INC, Warmington, PA, USA). Vectors were collected 48 h after transfection and filtered on 0.45 um filters before concentration with Vivaspin, 1,000,000 MWXCO PES (Sartorius Stedium Biotech). New batches of vector were prepared weekly. For each batch, the viral titer was determined before its use with HSC, by transduction of Jurkat cells, and stocked no longer than 1 week at 4 °C until required for its use for transduction of HSC. The titer was determined by transduction of Jurkat cells, using the spin-inoculation method following the same protocol used for transducing HSC, with serial dilutions of the concentrated viral preparations. The percentage of transduced cells was estimated 48 h after spin-inoculation by FACS analysis.

### Transductions

Transductions were carried out on variable amounts of cells (depending on their availability) and the corresponding appropriate number of viral vectors to reach the multiplicity of infection (MOI) indicated in each case, in a final volume of 1 mL of cell culture medium in the presence of hexadimethrine bromide (polybrene) at a final concentration of 8 μg/ml, by spinning at 800 g for 2 h at 32 °C (spin-inoculation). After transduction, the medium was removed the cells resuspended in fresh medium and transferred in 24-well plates at 37 °C with 5% CO2 for 48 h and then transplanted into zebrafish embryos. In some experiments, transducted-cells were kept in culture longer (up to 5 days) and the appearance of fluorescence analyzed by FACS and fluorescent microscopy (Fig. 1A).

### Zebrafish transgenic lines

Wild-type AB, *Tg(mpeg1:mCherry)* [35] and *Tg(mpx:eGFP)/Tg(mpeg1:mcherry)* [49] zebrafishs were maintained in compliance with the French Institutional Animal Care (CEEA-LR-13007). Embryos were kept in the presence of 1-phenyl-2-thiourea (PTU) to prevent melanin pigmentation [50] and staged as described by Kimmel and collaborators [51].

### Cell transplantation

Transduced cells were transplanted in the caudal vein (50-100 CD34 + cells and 50-200 Jurkat cells), in the YS (50-200 CD34 cells and 50–500 Jurkat cells), in the brain ventricle (100–200 Jurkat cells) and in the swim bladder (200–500 Jurkat cells) of Wild-type AB and Tg(*mpeg1:mCherry*) zebrafish embryos at 30–36 h post fertilization (h.p.f.) stage using a CellTrame micromanipulator (Eppendorf).

### Macrophage chemical and genetic depletion

Macrophages were depleted by injecting 5 nl of Liposome encapsulated Clodronate (Lipo-C) into the zebrafish embryo caudal vein as described previously [38]. Embryos with L-clodronate aggregates causing vessel occlusions and shortcuts were discarded.

The sequence of PU.1 morpholino is 5 -GATATACTGATACTCCATTGGTGGT- 3 (Gene Tools) [52]. PU.1 morpholino was resuspended in morpholino buffer—12 mM KCl and 20 mM HEPES—for a 2 mM stock concentration, and ~2 nl were injected in 1–4 cell stage embryos using a micro-injector system (Tritech Research Inc.). Efficacy of this sequence in knocking down PU.1 was validated by RT-PCR performed on embryos collected 24 h after PU.1 morpholino injection (Fig. S1).

### Microscopy

Embryos were anesthetized with tricaine (0.016%) and mounted on a glass cover dish with 0.7% low melting agarose (lateral views, rostral to the left). Fluorescence and Time-lapse images were acquired using Zeiss LSM510 at 20X or 40X magnification (Fig. 3, Video 2, 4, 5 and 6) or Zeiss AxioImager (Figs. 2A–H, 3B–G, 4, 5A, B, 5D, E, S2, S3, S4, S5, S6, and Video 1 and 3). Temperature was maintained at 28 °C by placing the dish in a temperature-control chamber during time-lapse acquisitions.

### Statistical analysis

Values are represented as mean ± SD. GraphPad Prism 7 was used for unpaired Student’s *t*-test to compare two groups of means and generate *p*-values. We used standard designation of *p*-values throughout the figures (ns not significant or *p* ≥ 0.05; *p < 0.05; **p < 0.01; ***p < 0.001; ****p < 0.0001). Details of number of replicates are provided in the individual figure legends.

### Supplementary information

Supplemental Legends and Figures

Supplemental Experimental Procedures

Video 1. Survival, maintenance, and proliferation of human JK-GFP cells in the zebrafish embryo.

Video 2. Migration and proliferation of human JK-GFP cells in the zebrafish embryo.

Video 3. Circulation and disappearing of CD34-GFP human cells after injection in the zebrafish embryo.

Video 4. Fragmentation of JK-GFP injected cells.

Video 5. JK-GFP cells phagocytosis by zebrafish embryonic macrophages.

Video 6 CD34-GFP cells phagocytosis by zebrafish embryonic macrophages.

## Data Availability

All data supporting this study are presented in this published article and in its Supplementary information files.
