# Supplementary material for: Macrophage depletion overcomes human hematopoietic cell engraftment failure in zebrafish embryo
Source: Cell Death Dis. 2024 May 1;15(5):305. doi: 10.1038/s41419-024-06682-x (PMC11063059; doi:10.1038/s41419-024-06682-x)
Supplement: Supplementary file 1 — Supplemental Legends and Figures [file 41419_2024_6682_MOESM1_ESM.pdf]

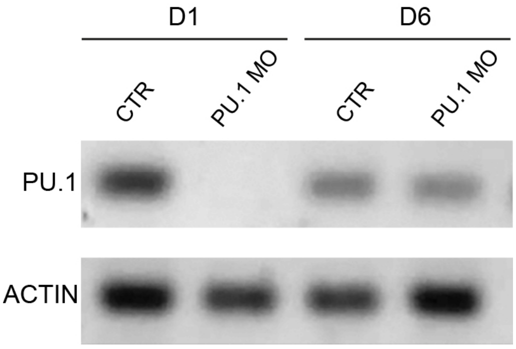

Figure S1. PU.1 morpholino validation.

Representative RT-PCR analysis displaying PU. 1 expression in zebrafish embryos injected with PU.1 morpholino (PU.1 MO) or morpholino buffer (MO Buffer). Analyses were performed at 1 (D1) and 6 (D6) days after injection. Actin is used as control.

PU.1 mRNA expression is knocked down at D1 in PU.1 MO-injected embryos. A rescue of PU.1 mRNA expression is visible at D6.

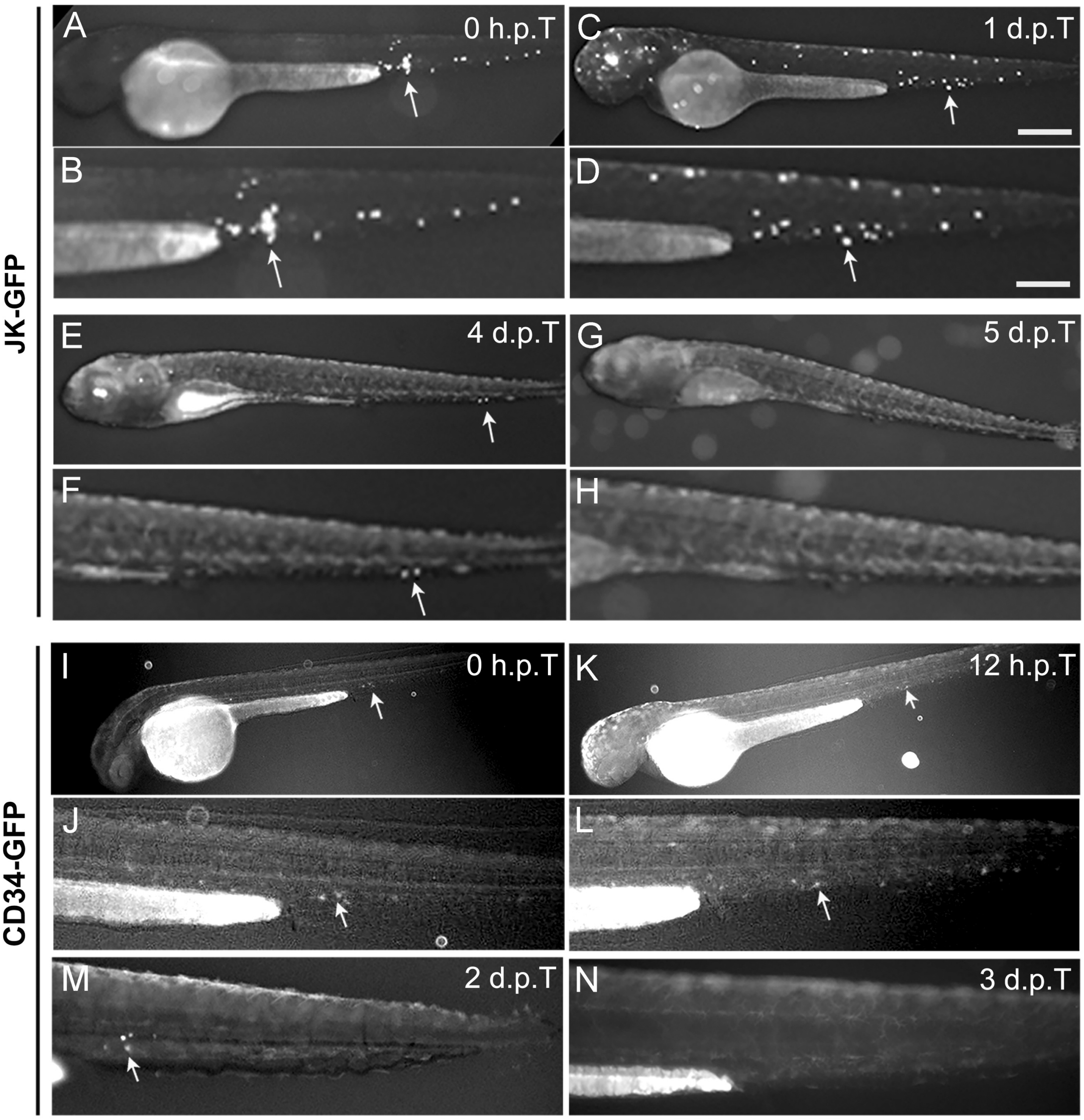

Figure S2. Circulation and disappearing of human cells injected in the zebrafish embryo. Transplantation of JK-GFP (**A-H**) or CD34-GFP (**I-N**) cells in the caudal vein of wildtype zebrafish embryos at 36 h.p.f. Imaging revealed cells mainly localized in the caudal hematopoietic tissue **(**arrows). JK-GFP at 0 h.p.T. (**A-B**, arrow), at 1 d.p.T. (**C-D**, arrow) and at 4 d.p.T. (**E-F**). JK-GFP disappeared at 5 d.p.T. (**G-H**) CD34-GFP at 0 h.p.T. (**I-J**, arrow) at 12 h.p.T. (**K-L**, arrow), and 2 d.p.T. (**M**) CD34-GFP disappeared at 3 d.p.T. (**N**) See **Video 5**. Scale bar: A, C, E, G, I, K: 250 µm; B, D, F, H, J, L, M, N:100 µm.

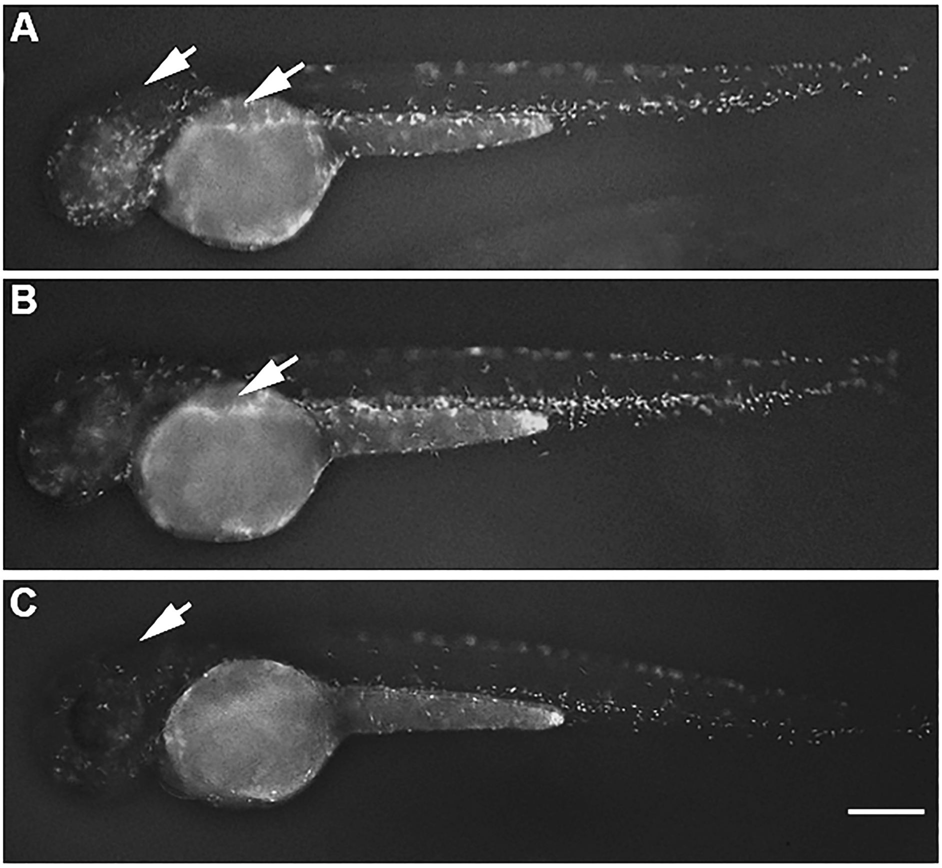

Figure S3. Effect of PBS injection on macrophage recruitment.

Imaging of *Tg(Mpeg1:mCherry)* zebrafish embryos at 48 h.p.f, 6 hours post injection of PBS into the swim bladder (**B**) and hindbrain ventricle (**C**), reveals a lack of macrophage accumulation in both injected sites compared to a non-injected *Tg(Mpeg1:mCherry)* zebrafish embryo (**A**). Arrows indicate the injection sites. Scale bar: 250μM.

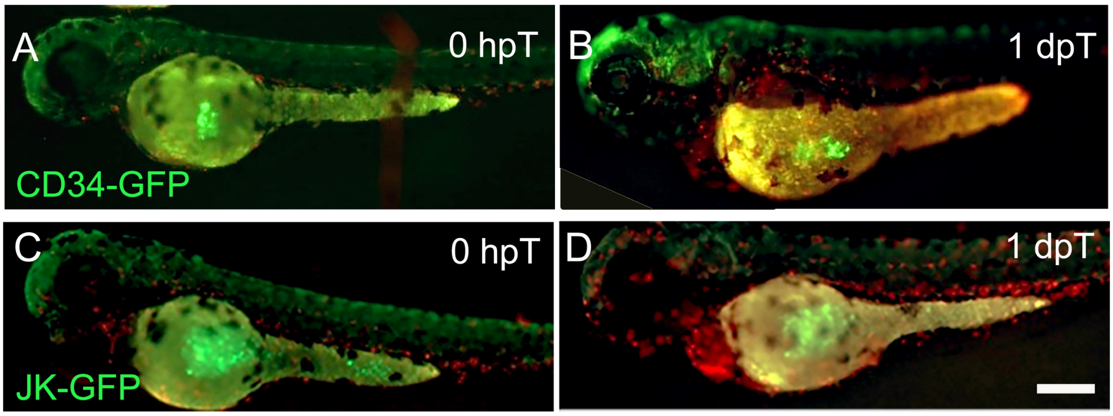

Figure S4. Injection of human cells in the yolk sac of the zebrafish embryo.

(**A** and **C**) Injection of JK-GFP or CD34-GFP cells respectively in the yolk sac of 30 h.p.f. *Tg(Mpeg1:mCherry)* zebrafish embryos. (**B** and **D**) No macrophages were detected into the yolk sac 1 d.p.T. JK-GFP and CD34-GFP cells populations were still present into the YS 1d.p.T(**C-E**). Scale bar: 50 μm.

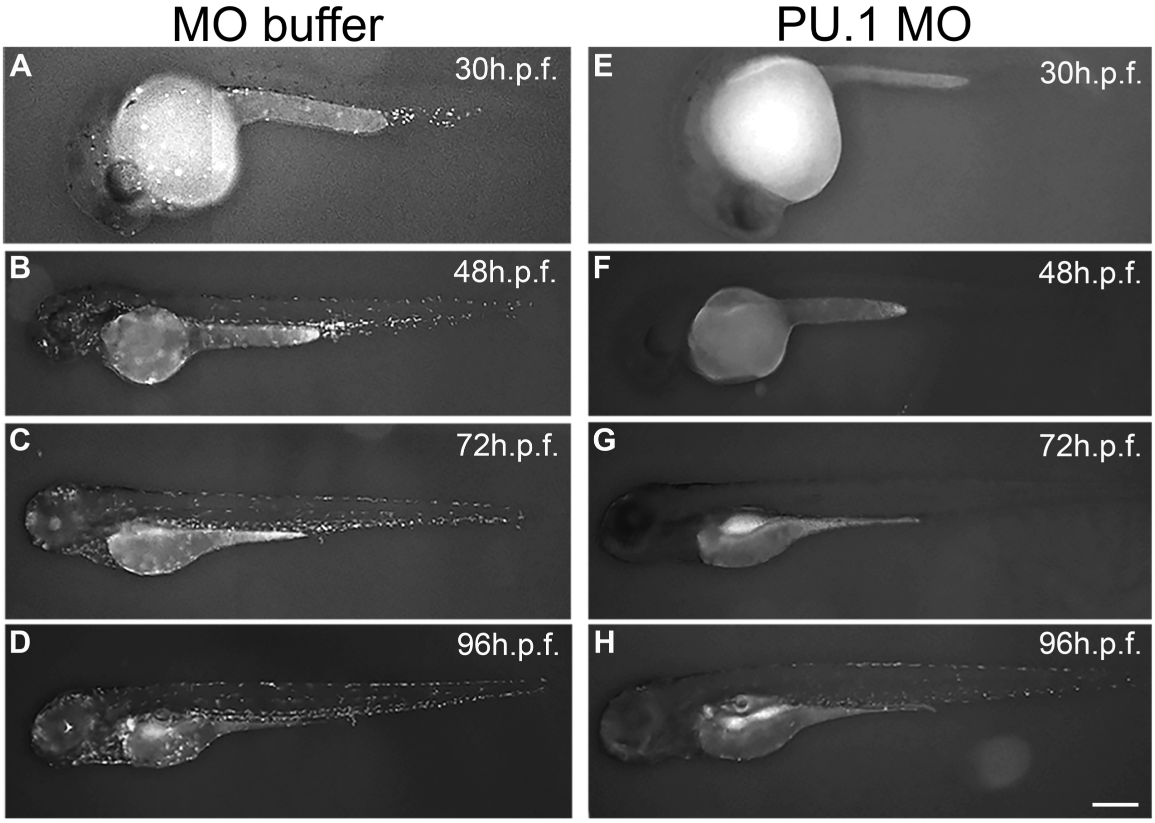

Figure S5. Macrophage depletion in PU.1 morphant embryos and rescue.

(**A-D**) Fluorescence imaging with a binocular loupe shows the macrophage emergence and colonization of *Tg(Mpeg1:mCherry)* embryonic tissues at 30 h.p.f. (**A**), 48 h.p.f . (**B**), 72 h.p.f. (**C**), and 96 h.p.f (**D**). PU.1 morphant embryos exhibit an absence of macrophages until 3 d.p.f. (**E-G**) and the reappearance of the first macrophages starting 4 d.p.f. (**H**). Scale bar: 250µM.

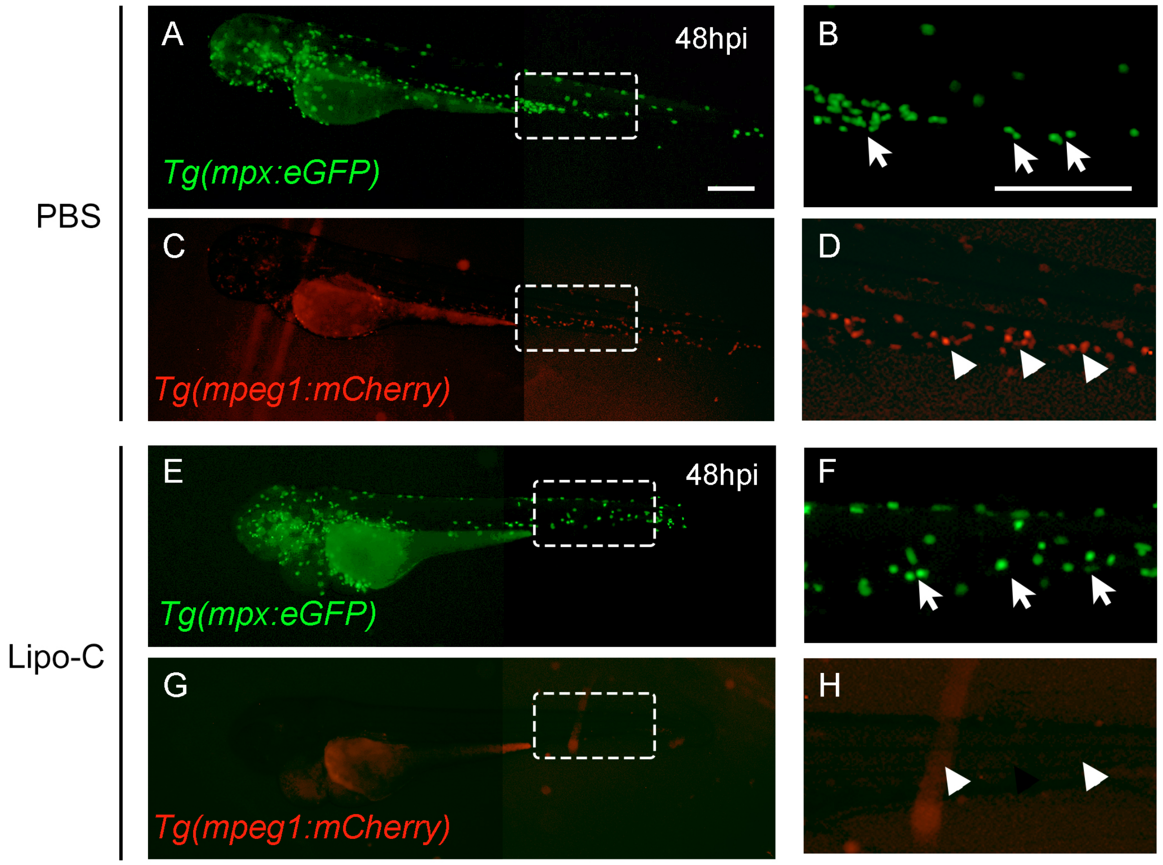

Figure S6. Macrophage depletion by L-clodronate has no toxic effect on neutrophil emergence.

Double transgenic Tg(mpx:eGFP)/Tg(mpeg1:mCherry) embryos at 32 h.p.f. were treated with PBS (**A-D**) or L-clodronate (Lipo-C) (**E-H**). Green fluorescence labels neutrophils and red fluorescence labels macrophages. Lipo-C treatment specifically depleted macrophages (white arrowheads) but not neutrophils (white arrows). The dotted regions in the figures on the left are enlarged in the figures on the right. Scale bar: 250 μm.

Video 1. Survival, maintenance, and proliferation of human JK-GFP cells in the zebrafish embryo.

Live imaging of human JK-GFP cells fate injected in a WT zebrafish embryo at 36 h.p.f. (1 frame/3 min). Real-time imaging at 4-, 6- and 7 days post transplantation, video in the top, middle and bottom position, respectively.

Video 2. Migration and proliferation of human JK-GFP cells in the zebrafish embryo.

Time-lapse imaging of human JK-GFP migration (asterisk) and proliferation (arrow) (Time code 00:15 and 00:24, arrow) in the CHT. Confocal microscopy (1 frame/3 min). Each frame is a maximum projection of 3 planes apart 1 µm. Scale bar: 50 µm. Time is indicated in minutes.

Video 3. Circulation and disappearing of CD34-GFP human cells after injection in the zebrafish embryo.

Time-lapse imaging of CD34-GFP cells after their injection in a zebrafish embryo at 36.5 h.p.f. in the caudal hematopoietic tissue (CHT) and during12.5 hours (36.5 to 48h.p.f., 1 frame/3 min). Arrows show CD34-GFP cells circulating in the CHT. Time is indicated in hours.

Video 4. Fragmentation of JK-GFP injected cells.

Time-lapse imaging of JK-GFP cells injected in a zebrafish embryo at 30 h.p.f. Each frame is a maximum projection of 3 planes apart 1 µm. Starting from 33 min some green followed cells are fragmented (arrow). Inset reveals a high magnification of fragmented cells (arrow) Scale bar: 25 µm. Time is indicated in hours and minutes.

Video 5. JK-GFP cells phagocytosis by zebrafish embryonic macrophages.

Time-lapse imaging of mCherry-expressing macrophage behavior in a zebrafish embryo at 2 d.p.T. injected with JK-GFP cells reveals the accumulation of macrophages around green cells and their phagocytosis (1 frame/3 min). Each frame is a maximum projection of 5 planes apart 0.8 µm. Scale bar: 20 µm. Time is indicated in minutes.

Video 6 CD34-GFP cells phagocytosis by zebrafish embryonic macrophages.

Z-stack confocal imaging of mCherry-expressing macrophage behavior in a zebrafish embryo 2 h.p.T of CD34-GFP reveals, in the tail region, the accumulation of macrophages around green cells and their phagocytosis (arrows). The z-stack is composed of 6 confocal planes spaced at 0.8 µm.
