# Supplementary material for: Macrophage depletion overcomes human hematopoietic cell engraftment failure in zebrafish embryo
Source: Cell Death Dis. 2024 May 1;15(5):305. doi: 10.1038/s41419-024-06682-x (PMC11063059; doi:10.1038/s41419-024-06682-x)
Supplement: Supplementary file 2 — Supplemental Experimental Procedures [file 41419_2024_6682_MOESM2_ESM.pdf]

Supplemental Experimental Procedures

Image processing and green-fluorescent quantification.

Fate of JK-GFP and CD34-GFP was quantified as previously described [1]. In brief, the quantification of green-fluorescent pixels was performed on the caudal hematopoietic tissue of each embryo after JK-GFP and CD34-GFP cells injection in the blood stream or in the yolk sac.

Images of embryos were processed using ImageJ software. Images were binarized using the automatic 'Threshold' plugin and the resulted signals were analyzed and measured using the 'Set Measurements' plugin.

PU.1 morpholino validation.

For PU.1 MO validation, pools of 10 to 20 zebrafish embryos injected with PU.1 MO or morpholino buffer were collected 1 and 6 d.p.i. and RNA was isolated with RNeasy (Qiagen). RNA concentrations and purity were assessed by measuring absorbance at 260 and 280 nm on the NanoVue spectrophotometer (GE Healthcare Life Sciences). RNA quality was analyzed using Agilent RNA 6000 Nano LabChip kit with the Agilent 2100 Bioanalyzer (Agilent Technologies). 20 ng of total RNA were reversed transcribed in a total volume of 20 µL using SuperScript III Reverse Transcriptase (Invitrogen) following the manufacturer's instructions. PCR was performed with Taq DNA polymerase (Invitrogen) in a total volume of 25 µL with 4 µL of reverse transcription reaction. The reaction conditions were 94°C for 30 seconds, 52°C for 1 min, 72°C for 1 min, for 25 cycles for PU.1 RNA and 20 cycles for Actin. The PCR products were analyzed by 2% agarose gel electrophoresis and visualized under UV illumination.

Primer sequences were: PU.1 5 -CAGAATGGAGGGGTACAT-3 and 5 -CGTTCTGACTGTCATCAA-3; actin 5 -TGGCATCACACCTTCTAC-3 and 5 -AGACCATCACCAGAGTCC-3 [2]. PCR product sizes were PU.1, 200 bp; actin, 221 bp.

Supplemental references

1. Travnickova J, Tran Chau V, Julien E, Mateos-Langerak J, Gonzalez C, Lelievre E, *et al.* Primitive macrophages control HSPC mobilization and definitive haematopoiesis. *Nature communications* 2015, **6:** 6227.
2. Lieschke GJ, Oates AC, Paw BH, Thompson MA, Hall NE, Ward AC*, et al.* Zebrafish SPI-1 (PU.1) marks a site of myeloid development independent of primitive erythropoiesis: implications for axial patterning. *Developmental biology* 2002, **246**(2)**:** 274-295.
